# Risk preferences impose a hidden distortion on measures of choice impulsivity

**DOI:** 10.1371/journal.pone.0191357

**Published:** 2018-01-26

**Authors:** Silvia Lopez-Guzman, Anna B. Konova, Kenway Louie, Paul W. Glimcher

**Affiliations:** 1 Center for Neural Science, New York University, New York, United States of America; 2 Institute for the Study of Decision Making, New York University, New York, United States of America; Middlesex University, UNITED KINGDOM

## Abstract

Measuring temporal discounting through the use of intertemporal choice tasks is now the gold standard method for quantifying human choice impulsivity (impatience) in neuroscience, psychology, behavioral economics, public health and computational psychiatry. A recent area of growing interest is individual differences in discounting levels, as these may predispose to (or protect from) mental health disorders, addictive behaviors, and other diseases. At the same time, more and more studies have been dedicated to the quantification of individual attitudes towards risk, which have been measured in many clinical and non-clinical populations using closely related techniques. Economists have pointed to interactions between measurements of time preferences and risk preferences that may distort estimations of the discount rate. However, although becoming standard practice in economics, discount rates and risk preferences are rarely measured simultaneously in the same subjects in other fields, and the magnitude of the imposed distortion is unknown in the assessment of individual differences. Here, we show that standard models of temporal discounting —such as a hyperbolic discounting model widely present in the literature which fails to account for risk attitudes in the estimation of discount rates— result in a large and systematic pattern of bias in estimated discounting parameters. This can lead to the spurious attribution of differences in impulsivity between individuals when in fact differences in risk attitudes account for observed behavioral differences. We advance a model which, when applied to standard choice tasks typically used in psychology and neuroscience, provides both a better fit to the data and successfully de-correlates risk and impulsivity parameters. This results in measures that are more accurate and thus of greater utility to the many fields interested in individual differences in impulsivity.

## Introduction

Time preference, or the preference of typical humans for immediate over delayed rewards, has long been a subject of study in economics, finance, neuroscience, and psychology. Temporal discounting describes this preference mathematically by quantifying how the subjective value of a payoff decreases as the time to its receipt increases. This delay-dependent decrease in subjective value is captured in models with diverse functional forms, with time preference typically summarized as a *discount rate or discount parameter*. The two most widely used functional forms are the *exponential* and the *hyperbolic* classes of models. Exponential discounting is derived from economic theory and assumes a constant rate of discounting at every time period. Hyperbolic forms are favored in psychology and neuroscience, as they can fit empirical data better [1, 2], exhibiting steeper discounting for near-future outcomes and shallower discounting of far-future payoffs, an inconsistency referred to as “present bias” [3]. More recently, psychologists have related this time preference to the multidimensional construct of impulsivity [4, 5]. Scholars of impulsivity have converged to a taxonomy that divides it into *action* and *choice impulsivity*, and officially consider the measurement of discounting as a quantitative assessment of the latter [6]. We note that for economics, impulsivity is not directly equated to elevated discounting. For this field, when discounting is constant (such as in exponential discounting) the *discount rate* reflects consistent impatient preferences. By contrast, in hyperbolic discounting the *discount parameter* may reflect inconsistencies in these preferences (present bias) and therefore reflect what economists consider impulsivity. In this paper, we attempt to reconcile these insights from economics and bring them to the attention of fields like psychology, neuroscience, and psychiatry.

With hundreds of publications a year focused on delay discounting, these measurements have now been performed on a wide variety of healthy and clinical populations under many different conditions. Examples of the prevalence of this measure in the literature include large studies and meta-analyses in healthy volunteers [7–13], as well as case-control studies in a variety of patient populations with substance use disorders [14–16], anorexia [17], obesity [18], personality disorders [19], ADHD [20], and anxiety [21–23]. Adding to this growing literature, several groups have investigated the neural basis of temporal discounting [24–29] and others have explored the effect of behavioral or neural manipulations on temporal discounting in healthy volunteers [30–33].

In parallel to using measures of temporal discounting to assess impulsivity, there has been growing interest in the quantitative assessment of risk attitudes by measuring formal risk preferences, that is, an individual’s general proneness to or avoidance of risky prospects, in a wide array of populations [34–37]. In expected utility theory and many other cardinal economic theories of choice, risk attitude is associated with the curvature of the utility function. This function can be interpreted as the mapping from the objective amount of a good (or money) to the subjective value derived from obtaining it. In these theories, this function is related to choices over probabilistic outcomes [38, 39]. When a subject’s utility function is linear, she chooses between lotteries as if maximizing *expected value* (choosing the option for which the product of the value of the prize and its probability is highest); this is often referred to as risk neutrality. When the utility function is concave, subjects are risk averse; when it is convex, subjects are risk seeking. Many studies have concluded that there is great diversity in risk preference and that risk attitudes are affected by age, context and even physiological states like menstrual cycle phase [40–45].

More recently, economists have examined how risk attitudes might introduce possible confounds to the empirical measurement of discount rates [46–48]. The key ideas in this literature are: first, that because the future is inherently uncertain, risk attitude must play a role when evaluating future prospects irrespective of their time preferences [49, 50]; and second, that the preference for smaller-sooner rewards may be driven by either impatience (what in psychology is considered the “choice impulsivity” dimension of impulsivity) or by diminishing marginal utility as captured by nonlinearities in the utility function [51, 52]. Although accounting for risk preferences when estimating discount rates is of growing importance in the economics and management fields, with few exceptions [26, 53], it is not a practice that has fully impacted studies in the neuroscience and psychology fields, even when both types of preferences are measured in the same individual. Further, because in economics the focus is rarely on the employment of these measures for the study of individual differences, the size of the impact of risk preference on individual discount rate estimates has not been well characterized.

In this study, we investigated how individuals’ risk attitudes bias the estimation of their discount rates. As our goal is to propose this methodology to the fields that do not already employ it, we used simple standard binary choice tasks widely used in the psychology and neuroscience literature [24, 25, 54, 55] in a real-world non-expert sample. We hypothesized that a procedure that estimated individual subject temporal discounting rates, but that also incorporated independently estimated risk attitude parameters, would outperform standard (economic) tools for estimating temporal discounting rates. We found that in our community sample of subjects, where there was a wide diversity of individual risk preferences, our approach showed superior performance in capturing individual intertemporal choice behavior. Unlike previous studies that employed similar methods but that focused on population-level discount rate estimates, here we focused on individually estimated parameters. We found that the standard approach introduces a systematic pattern of bias that distorts individual discount rate estimates. We conclude that ignoring individuals’ risk attitudes when measuring temporal discount rates can significantly impact interpretations about their degree of choice impulsivity.

## Materials and methods

### Subjects

All participants gave written informed consent in accordance with the procedures of the University Committee on Activities Involving Human Subjects of New York University and the Institutional Review Board of the New York University School of Medicine, which approved this study. We recruited 56 medically healthy participants (11 women) from the general community (via flyers, internet advertisement and word-of mouth) without significant history of substance use or psychiatric illness. Subjects’ demographic information including average education level, income level, employment and race and ethnicity breakdown is presented in Table 1.

**Table 1:** Sample demographics.

Gender (% males)	73.3%
Age (years)	44.04 (12.4)
Nonverbal I.Q.	91 (2.5)
Education (years)	13.8 (2.0)
Unemployed (%)	14%
Income (monthly $)	1770.49 (263.73)
Race (% C—% AA)	47.6—52.4
Ethnicity (% Hispanic)	14.3

Values are presented as mean (± 1 standard deviation) unless they are percentages. Race is divided into Caucasian (C) and African American (AA). No other races were present in our sample. Ethnicity is divided into Hispanic and non-Hispanic. The education and income of our sample match median education level and personal income level adjusted for educational attainment in the United States.

### Session description

After collecting pertinent contact and demographic information, subjects completed the intertemporal choice task and the risk task. The order of the tasks was randomized across subjects and sessions. Both tasks were computerized (Psychtoolbox for MatLab and e-prime 2.0) and were completed in a private testing room. Subjects were given extensive instructions as well as some practice trials to ensure they understood the tasks fully before beginning. Subjects completed 2 sessions separated by at least one week.

### Risk attitude (RA) task

The task consisted of 64 lottery choices in the gain domain. Each trial involved a choice between a fixed amount of money ($5 for sure) and a lottery with the probability level associated with winning a (usually higher) amount changing from trial to trial. Each lottery had two possible outcomes: $*v* or $0. The exact amounts of *v* were: $5, $6, $7, $8, $9, $10, $12, $14, $16, $18, $20, $23, $26, $30, $34, $39, $44, $50, $57, and $66. We used three winning probabilities, *p*, 25%, 50%, and 75%. In this case, each lottery can be fully described by *v* and *p*. Each amount *v* was presented with each probability level once in random order over 4 blocks of 16 trials. In addition, 4 “catch” trials were included at the start of each block. These trials always presented a choice between $5 for sure vs. 50% chance of $4 or $0. Thus, these trials in addition to trials that offered risky lotteries where *v* was $5 (10 in total), allowed us to assess the frequency of first order stochastic dominance violations, that is, whether subjects chose the objectively worse of the two options. We considered two or more of such violations as evidence that we could not reliably model subjects’ choices with a monotonic utility function. Both the fixed $5 and the lottery were presented side by side on the screen. Subjects were told that each lottery image represents a physical paper bag that contains 100 poker chips, some red and some blue. Subjects were told the precise number of red and blue chips in the bag by explicitly showing the number and by coloring parts of the image according to the proportion of red and blue chips.

### Intertemporal choice (ITC) task

The task was a two alternative forced choice task consisting of 102 trials that presented two options, one monetary reward to be received on that day and one monetary reward to be received with variable delay (in days). On each trial, both the fixed immediate and delayed options were presented side by side on the screen. The range of rewards across both periods went from $2 to $66. The immediate reward was either $2, $5 or $15, and the delayed reward was always a larger amount in the following increment levels: for trials with $2 immediate reward, +$5, +$10, +$20, +$40, +$64; for trials with $5 immediate reward, +$5, +$10, +$20, +$40, +$60; and for trials with $15 immediate reward, +$5, +$10, +$20, +$40, +$50. The actual delayed alternative presented was the result of the exact given increment level or plus or minus $1. Possible delays were 5, 10, 30, 60, 90, 120 and 150 days. The actual delay presented corresponded to the stated delays in days or plus or minus one day. For example, one of the trials was a choice between $5 today and $66 in 89 days. The selected choice set allowed for a very distributed investigation of the space to ensure our ability to estimate very high or very low discount rates with equivalent precision.

### Incentive compatibility and payment

We compensated subject participation with a $10 fee and a bonus. At the end of the session, one choice from either the ITC or RA task was randomly selected to determine this bonus. This ensures that subjects’ decisions were incentive compatible: they do not know which choice will count so their best strategy is to treat each one as if it were the one that counts. Payment for both the participation fee and the bonus was made via money order in the following way: subjects received a code via a text message to their phone on the day the payment was due, to prevent against subjects forgetting payments, to claim their bonus at their convenience. Critically, because all payments were made this way we introduced no differences in the transaction costs for different types of payments (participation fee, RA task payment, ITC immediate payment or ITC delayed payment). For ITC delayed payments, subjects received the code on the date corresponding to the delay for that chosen option.

### RA task analysis

To quantify technical risk attitude for each session, we used a power utility model to fit the choice data from the RA task as we have described previously [40, 41, 43, 54, 56]. In this model, the utility (*U*) of each option (safe or lottery) is given by:

$$\begin{array}{c} U_{\left(option\right)}=pv^{\alpha } \end{array}$$ (1)

where *v* is the dollar amount, *p* is the probability of winning, and *α* is the curvature of the utility function which serves as a subject-specific measure of technical risk attitude. A subject whose *α* = 1 has a linear utility function and is thus risk neutral. A subject whose *α* > 1 has a convex utility function and is thus risk-seeking (reflecting a tolerance of risk and uncertainty). A subject whose *α* < 1 has a concave utility function and is thus risk-averse (reflecting a distaste for risk and uncertainty). Using maximum likelihood estimation in MATLAB, we fit a single logistic function to the trial-by-trial choice data of each subject:

$$\begin{array}{c} Pr_{\left(lottery\right)}=1\;/\;\left(1+e^{-\gamma (U_{\text{lottery}}\;-\;U_{\text{safe}})}\right) \end{array}$$ (2)

where *Pr* is the probability that the subject chose the lottery option on a given trial, *U*_safe_ and *U*_lottery_ are the utilities (subjective values) of the safe and lottery options, respectively, and *γ* is the slope of the logistic function, which is a second subject-specific parameter. The parameter *γ* captures the stochasticity (as it is related to the randomness of the choice data).

### ITC task analysis

We applied four models to quantify subject time preferences. The first model was a non-normative hyperbolic discounting model [57], which assumes an underlying linear utility. We call this model Linear utility Hyperbolic discounting (LH) for ease of reference in the text. In this model, the utility (*U*) of each option (immediate or delayed) is given by:

$$\begin{array}{c} U_{\left(option\right)}=v\;/\;\left(1+\kappa d\right) \end{array}$$ (3)

where *v* is the dollar amount of the option, *d* is the delay to the delivery of *v* (which is 0 for the immediate option), and *κ* is the discount parameter which serves as a subject-specific measure of impulsivity. The second model was a non-normative hyperbolic discounting model that took into account the curvature of the utility function as estimated by Eq (1), captured in the parameter *α*. We call this model Nonlinear utility Hyperbolic discounting (NLH) for ease of reference in the text. In this model, the utility (*U*) of each option is given by:

$$\begin{array}{c} U_{\left(option\right)}=v^{\alpha }\;/\;\left(1+\kappa d\right) \end{array}$$ (4)

The third model was a normative exponential discounting model which assumes an underlying linear utility. We call this model Linear utility Exponential discounting (LE) for ease of reference in the text. In this model, the utility (*U*) of each option is given by:

$$\begin{array}{c} U_{\left(option\right)}=ve^{-\kappa d} \end{array}$$ (5)

The fourth model was a normative exponential discounting model that took into account the curvature of the utility function again by using *α*. We call this model Nonlinear utility Exponential discounting (NLE) for ease of reference in the text. In this model, the utility (*U*) of each option is given by:

$$\begin{array}{c} U_{\left(option\right)}=v^{\alpha }e^{-\kappa d} \end{array}$$ (6)

Using maximum likelihood estimation in MATLAB, we fit a single logistic function to the trial-by-trial choice data of each subject:

$$\begin{array}{c} Pr_{\left(delayed\right)}=1\;/\;\left(1+e^{-\beta (U_{\text{delayed}}\;-\;U_{\text{immediate}})}\right) \end{array}$$ (7)

where *Pr* is the probability that the subject chose the delayed option on a given trial, *U*_immediate_ and *U*_delayed_ are the utilities (subjective values) of the immediate and delayed options, respectively, and *β* is the slope of the logistic function, which is another subject-specific parameter. The parameter *β* captures the stochasticity (as it is related to the randomness of the choice data).

### Model comparison

The models were evaluated by comparison of their cross-validated log likelihoods: we computed the log likelihood by leave-one-out cross-validation, fitting the model to the data from all the trials except for one. This process was repeated iteratively for each of the trials and the likelihoods were added to compute the final log likelihood. We chose this leave-one-out method to avoid discarding too much data from the estimation process as there were no replicates of the trials and the indifference point location could be hard to resolve for some sessions (see S1 Fig for a description and rationale of the choice set.)

## Results

**Fig 1:**
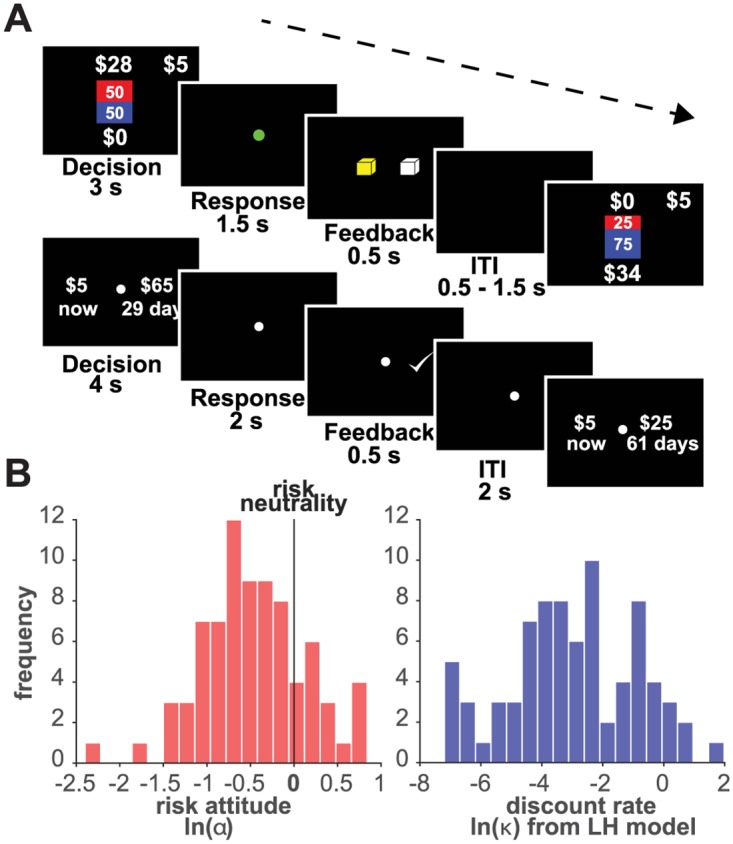
RA and ITC task design. A (top): RA task design, the safe and lottery options are simultaneously displayed during the decision phase. A green dot cues the response time. A yellow square provides feedback on the choice entered. A variable inter-trial interval (ITI) follows. A (bottom): ITC task design, the immediate and delayed options simultaneously displayed during the decision phase. The offer disappears and a white dot cues the response time. A white check mark provides feedback on the choice entered. A variable ITI ensues. For a description of the choice set (see S1A and S1B Fig). B (left): distribution of natural logarithm of risk attitude parameter (ln(*α*)) across all subjects and all sessions. B (right): distribution of natural logarithm of discount parameter (ln(*κ*)) estimated from the LH model across all subjects and all sessions.

By design, our subjects exhibited a wide demographic diversity and were representative of the general urban population (see Table 1). None of our subjects were students or had any advanced knowledge of finance or previous experience with the type of tasks used in this study. Subjects performed two decision-making tasks during each session, an intertemporal choice (ITC) task and a risk attitude (RA) task (see Fig 1A). We recruited 56 subjects to complete 2 identical testing sessions to allow us to assess test-retest reliability. We found that for our tools and subject sample, reliability was high (intraclass correlation coefficient for risk attitude and discount parameters *r* > 0.54, *p* < 0.05). For our analyses, we excluded any sessions for which the goodness-of-fit (*R*^2^) for the risk attitude parameter estimation was lower than 0.4. We also excluded sessions in which subjects *always* selected one of the options in the ITC task as this makes it impossible to resolve their indifference point with our choice set. After these exclusions, we analyzed a total of 78 sessions (2 sessions for each of 39 subjects). In most of the following analyses, each session is regarded as a separate data point. Since we do not perform group comparisons between subjects (all comparisons are of different model fits within subjects), we note that there are no statistical implications from treating the data in this manner.

### Diversity in risk preferences

We found that our sample exhibited very diverse risk preferences. We fit the power utility model shown in Eq (1) to the RA task choice data from each session using the softmax decision rule in Eq (2). We estimated a subject and session-specific RA parameter (*α*). This parameter ranged from 0.1 to 2.173, as shown in Fig 1B (note that values in those distributions are natural log-transformed). The average RA parameter (*α*) was 0.7508, and the median was 0.6087. Note that although on average our sample exhibited risk aversion (*α* < 1), individual risk preferences were heterogenous: there was a wide range of *α* values with many subjects deviating far from risk neutrality (*α* = 1, or 0 in the log space shown in Fig 1B).

### Diversity in time preferences

To evaluate the diversity of time preferences in our sample, we first ignored risk preferences and estimated discount parameters using standard methods (effectively assuming risk neutrality in all subjects.) To do so, we fit the “Linear utility Hyperbolic discounting” model (LH) shown in Eq (3) to each session’s ITC choice data using the softmax decision rule in Eq (7). We estimated a subject and session-specific ITC discount parameter (*κ*). The distribution of subject discount parameters is shown in Fig 1B (note that values in those distributions are natural log-transformed). Values ranged from 0.001 (equivalent to the discounting of 2.9% of the reward’s value after a delay of one month) to 6.4 (equivalent to the discounting of 99.5% of the reward’s value after one month). The mean *κ* was 0.3139 and the median was 0.0499 (equivalent to the loss of 60% of the reward’s value after one month).

### Taking risk preferences into account improves the fit to ITC choice data

We used maximum-likelihood estimation to fit four different models to each individual session ITC choice data: two hyperbolic discounting models and two exponential discounting models. We focused on these two classes of models because they are the most prevalent in the literature. Exponential discounting provides a normative account of discounting grounded in discounted utility theory. Hyperbolic discounting often provides a better fit to behavioral human and animal data [3, 58]. Of the two hyperbolic models, one did not take risk attitude into account (assumed a linear utility function, LH) as in Eq (3) and the other (NLH) used the estimated risk attitude (the utility function curvature parameter *α* from Eq (1)) as in Eq (4). Similarly, for the exponential type, one model assumed a linear utility function (LE) as in Eq (5), and the other (NLE) used the *α* as shown in Eq (6). To evaluate these four models, we compared their cross-validated log likelihoods (LL). Note that all four models have the same number of free parameters because the *α* parameter in the models that incorporated a risk attitude estimate (NLH and NLE) was fixed, taken from the maximum likelihood estimation procedure performed on independent data from the RA task (Eq (1)). We employed cross-validation to avoid over-fitting by iteratively fitting the model on all trials but one and computing the log likelihood of the model for the left-out trial (see Materials and methods section).

**Fig 2:**
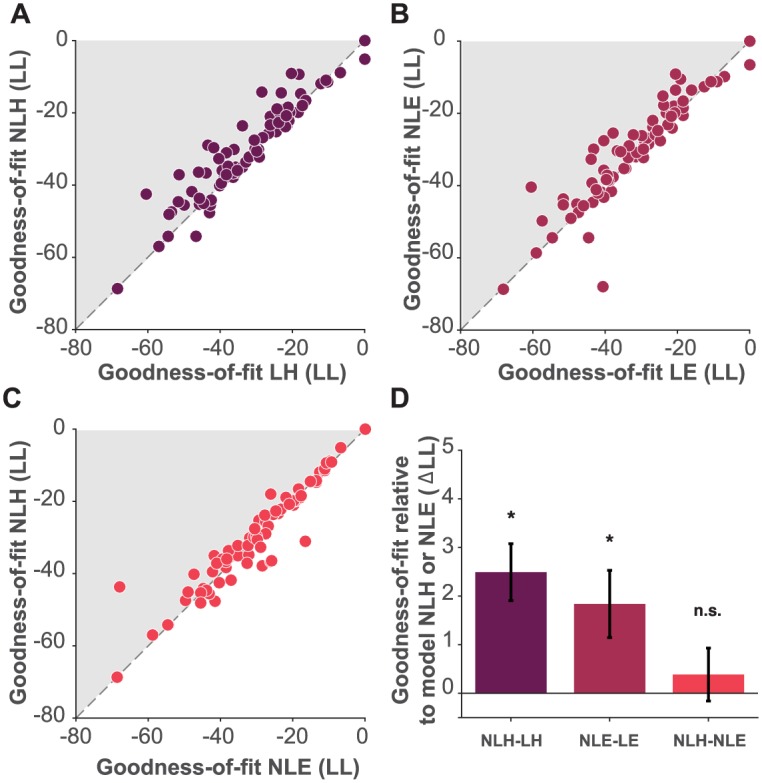
Model comparison. A: cross-validated log likelihood (LL) comparison of model LH against model NLH. Each dot corresponds to data from a single subject’s session. B: LL comparison of model LE against model NLE. Panel C, LL comparison of model NLE against model NLH. Shaded areas for panels A and B correspond to sessions for which the nonlinear utility models fit the data better than the linear utility models. C: Shaded area corresponds to sessions for which the NLH model fit the data better than the NLE model. D: average difference in LL across all sessions between model NLH and model LH (dark color), between model NLE and model LE (intermediate color) and, between model NLH and model NLE (light color), black bars indicate S.E.M.

We found that the LL was higher for the model that included an independent estimate of risk when estimating a hyperbolic discount parameter (NLH) than for the model that did not (LH), for the majority of the sessions in our data set (Fig 2A). Similarly, we found that the model that included an independent estimate of risk when estimating an exponential discount parameter (NLE) had a higher LL than did a model that omitted this term (LE) for the majority of the sessions in our data set (Fig 2B). Interestingly, the two models that included an estimate of risk attitude (NLH and NLE) performed similarly well, suggesting that in our data goodness-of-fit does not rely on exponential or hyperbolic assumptions (Fig 2C). AIC and BIC scores were also compared and yielded similar results to our cross-validated LL comparison (see S2 Fig). We note that for both the hyperbolic and exponential forms, the advantage in goodness-of-fit of the models that account for risk (NLH relative to LH and NLE relative to LE) was correlated with the discount parameter. However, this correlation was not only carried by outliers as the rank-ordered Spearman coefficient was significant, rho = 0.5, *p* < 0.001 for the exponential form and rho = 0.52, *p* < 0.001 for the hyperbolic form. This means that the advantage of NLE and NLH was not only true for impatient outliers.

To evaluate the overall performance of the models, we computed the average difference in LL across all sessions and subjects of the LH, LE, and NLE models relative to the LL of the NLH and NLE models (Fig 2D and for all possible comparisons between models see S3 Fig). Across all the data, the risk-incorporating models (NLH and NLE) show overall significantly superior performance than models that did not include risk (LH and LE). The difference in goodness-of-fit between NLH and NLE was not significant. However, when we simulated data generated from each model, NLE proved to be less distinguishable from the other models than NLH (see S2 Fig); the NLE model fits were equally good for data generated from NLE itself as for data generated from other models, whereas NLH fits were significantly better for data generated from NLH itself and not from the other models. We therefore focus on the NLH model’s superiority for the rest of our analyses.

### Failing to measure risk attitude systematically deviates estimates of discount parameter

**Fig 3:**
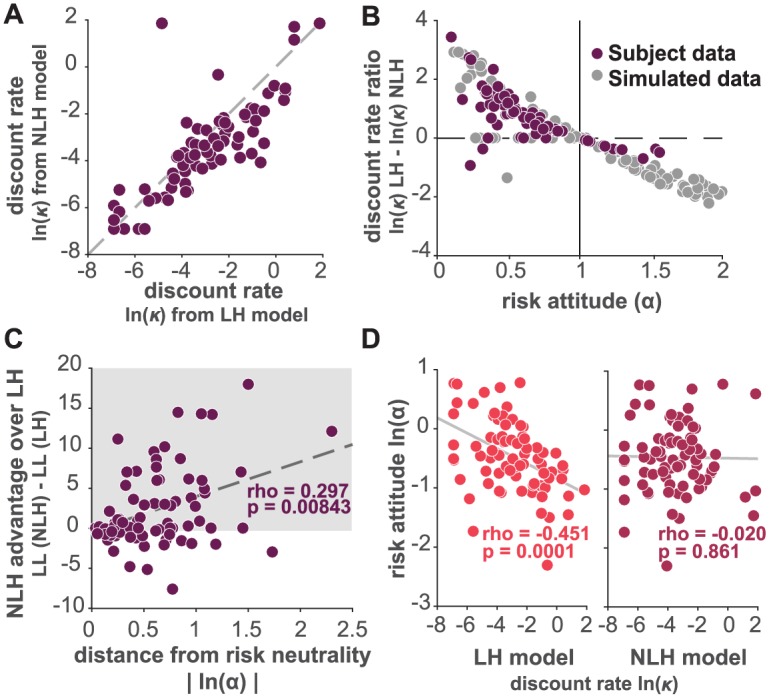
A systematic bias in discount parameters. A: comparison of estimated discount parameters from model LH against model NLH for each subject’s sessions presented as natural logarithm of discount parameter (ln(*κ*)). B: discount parameter bias computed as difference between the natural log of estimated parameters from model LH against model NLH (ln(*κ*)LH − ln(*κ*)NLH), plotted as a function of the corresponding risk attitude parameter (*α*), dark dots represent data from each of our subjects’ sessions, gray dots represent simulated data. C: difference of goodness of fit (LL from NLH—LL from LH) between NLH and LH model as a function of the absolute value of the natural logarithm of *α* (|ln(*α*)|), risk neutrality here is 0 and any value above 0 is either risk averse or risk seeking. Spearman correlation: rho = 0.297, *p* < 0.01. Shaded area corresponds to the sessions for which model NLH fit the data better than model LH. D: correlation between the natural logarithm of the risk attitude parameter (ln(*α*)) and the natural logarithm of the discount parameter and model NLH (dark color).

Having established that model NLH provides a better account of the data than models that do not incorporate risk attitude, we next examined the magnitude and direction of the misestimation when risk preference was ignored. We compared the individual discount parameter estimates (*κ*) obtained from model LH (the most commonly used model in the psychology literature) and those obtained with model NLH. We found that the two discount parameter estimates indeed differed for most of our subjects (Fig 3A): for the large majority of subject sessions the discount parameter from model LH was higher than the discount parameter from model NLH. We next tested whether the difference in estimated discount parameters depended systematically on risk preference (captured by the *α* parameter). We simulated 100 data sets using our NLH model with values for the risk parameter samples drawn from a uniform distribution within the range of our RA task choice set. This allowed us to cover the space of *α* fully, given that in our sample (as in most reported studied subjects in the literature) most subjects are risk averse (*α* < 1). We observed that the difference in discount parameter estimates obtained for these synthetic data sets (*κ* from LH—*κ* from NLH) led to a systematic shift in the estimated discount parameter as a function of the risk preference parameter (*α*) (see gray points in Fig 3B).

We also computed the difference in discount parameter estimates from both models for our subject data and saw the same trend we observed for simulated data (see dark points in Fig 3B): When the *α* parameter is less than 1 (risk aversion) the *κ* parameter estimated by LH is higher than that of NLH. *Thus, the standard method employed by most studies leads erroneously to the conclusion that risk-averse individuals are more impulsive than they really are*. Conversely, when the *α* parameter is higher than 1 (risk tolerant) the *κ* parameter estimated by LH is lower than that of NLH: *risk-seeking individuals appear to be less impulsive than they really are*. Logically, when *α* = 1 NLH and LH converge to the same functional form and the discount parameter estimates are identical. Further, the larger the deviation from risk neutrality, the more model NLH outperforms LH, suggesting that any individuals with non-neutral risk preferences are much better captured by our model NLH (Fig 3C).

*Importantly, the NLH model also resulted in the orthogonalization of the risk attitude parameter* *α* *and the discount parameter* *κ*, *while* *α* *and* *κ* *derived from model LH were significantly negatively correlated* (Fig 3D). For model LH, the Pearson correlation coefficient was r = -0.451, p = 0.0001. Conversely, the correlation between *α* and the *κ* derived from model NLH was not significant (Pearson’s r = -0.02, p = 0.861). Taken together, these results show that the NLH model yields a discount parameter that is not only more precise but also allows for *κ* and *α* to reflect distinct aspects of decision-making rather than being conflated as is the case with estimates derived from the traditional model LH.

## Discussion

We show here that 1) a temporal discounting model that incorporates an individual out-of-sample estimate for risk attitude outperforms models that assume risk neutrality (Fig 2), and 2) that failing to incorporate this risk attitude estimate leads to systematically biased discount parameters that entangle risk attitude and choice impulsivity (as in Fig 3); risk-averse subjects appear more impulsive than they are.

The finding that risk attitude interacts with intertemporal preference is not unexpected. Recent advances in economic research focusing on time preferences have been prompted by the potential confounding influence of non-linear utility upon estimates of discount parameter [59]. Studies devoted to this issue have applied either joint elicitation techniques of the kind used in our study (where risk preferences and time preferences are estimated from separate choice tasks) or methods where both utility and discounting are elicited from intertemporal choices [60, 61]. There is still no theoretical consensus among economists on the correct interpretation of the relationship between utility for risk and instantaneous utility for time [62] and, for some, subjective valuation of temporal payoffs may differ from that of risky ones [60, 63–65], but see [66]. However, from an empirical point of view, our study contributes to the growing number of reports that indicate there is a systematic bias in the discount parameter estimates when risk attitude is ignored. This dependence can be observed even in simple and widely employed binary choice tasks and at the individual subject level.

While we recapitulate the results reported by previous studies coming from economics and finance, the novel contribution of our work lies in two keys aspects: First, unlike previous studies we do not aggregate the subject data and estimate a single group parameter for the entire sample. Instead, we capitalize on the diversity of individual preferences by independently estimating a risk aversion parameter and a discount parameter for each subject’s session. This diversity is the result of our rich community sample which is representative of the average urban dweller. With some notable exceptions [51], the majority of previous studies on this topic have been performed with expert samples such as economics or finance students, which may lack the diversity in demographics that would allow for inferences about how these decisions are made by typical people. If not enough variance in risk preferences is achieved in the studied sample, it is possible the effect of risk may not be fully observable. We found wide variability in parametric risk attitudes in our sample, despite the mean not being much different from what has been previously reported in the literature. Furthermore, we simulated data with higher risk-seeking preferences to explore what the bias in the discount parameter would be in that direction. Although not often seen in healthy volunteers, risk-seeking behavior is more prevalent in psychiatric conditions [67–72], making this bias relevant for these types of studies. Second, we employed widely used binary choice tasks that can easily be completed by any subject and do not require any explicit knowledge of finance. We believe this is important if these assessments are to be deployed across different types of populations, including those without any higher education (in our sample the average education level is 13.8 years, see Table 1) and without any sophisticated understanding of interest rates and finance in general.

To establish whether the diversity in risk preferences has implications for how well models of temporal discounting fit individual subject choice data, we compared four models that are used in this growing literature. Two of these models assume that subjects are risk neutral: the linear hyperbolic (LH) and exponential (LE) models. The two other models incorporate subject-specific risk attitudes, that is, the curvature of the individual’s utility function. The LH model is the most widely used in the psychology and neuroscience literature to parametrically investigate discounting as a measure of impulsivity. It has been shown to fit behavioral data better than the normative exponential (LE) alternative, which often fails to fit especially highly impulsive subjects’ data. The superiority of LH over LE has usually been linked to the fact that subjects exhibit a “present bias”, that is, the tendency to give stronger weight to payoffs that are closer to the present time when considering trade-offs between two future moments. As such, subjects’ preferences have been reported to be better modeled by a hyperbolic rather than an exponential decay function. We recapitulated that result in our sample but found that our NLH model fit the intertemporal data better than both LH and LE across most of our subjects and sessions, suggesting taking risk into account is important regardless of where one sits in the exponential versus hyperbolic debate. Interestingly, NLH and NLE had similar performance. Consistent with previous studies [51], accounting for risk in the exponential form (NLE) resulted in a significantly better fit than the hyperbolic form that did not (LH) (see S3 Fig), which suggests that discounting becomes more constant when risk is considered. However, NLE seems to be a less precise model at distinguishing between LE and NLE generated data, while our model NLH was more specific (Fig 2 and S2 Fig). This means that NLE is too flexible, fitting data equally well even when it comes from different generative models. By contrast, NLH was a more selective model, a feature that may be more desirable for fields such as neuroscience that seek the true (neural) generative mechanism behind the behavior exhibited by the decision maker.

We have shown that ignoring risk attitude results in a systematic bias in the discount parameter estimates (Fig 3). For a large range of *α* values close to 1, LH and NLH do not perform differently, but the more risk averse or the more risk-seeking an individual, the more our approach outperforms the traditional approach. One clear implication of using the LH model in subjects that exhibit widely varying risk attitudes is that discount parameter estimates may be biased. We showed that when subjects are risk averse, the LH model returns a higher *κ* estimate value than NLH and conversely, when subjects are risk-seeking, LH underestimates the discount parameter. If one were comparing risk-averse individuals (e.g., the average healthy person) to less risk averse individuals (e.g., problem gamblers) the standard approach might lead to conclusions about differences in choice impulsivity between these groups that might be (perhaps better) explained by differences in risk attitude. Some studies have already begun to suggest that reported differences in discount rates between substance users and controls may be inflated by the use of the LH model [73]. This could also prove relevant for the comparison between patients with anxiety disorders (who have been reported to be highly risk-averse [74]) and healthy controls.

Of particular interest to us is the fact that investigations into the neural correlates of utility (or subjective value) and of intertemporal choice have also shown that the shape of this function is important when computing discounted subjective value [26]. We found that the NLH model not only fits behavioral data better but also is the most specific model (see S2 Fig), suggesting that the NLH model could be the best approximation to the brain’s generating model, a question that remains to be tested in future studies. This is relevant for any study that attempts to tie time preference behavior to neural activity. For instance, it has been suggested that temporal discounting could provide a behavioral marker for psychiatric diagnosis and prognosis [75, 76]. Transforming neural measurements of temporal discounting into a valid biomarker of clinical utility for psychiatry supposes that it accurately measures the intended biological and behavioral process [77, 78]. Our study shows that incorporating risk attitude is critical to delineating this process with precision.

As shown in Fig 2 (and S3 Fig) we found a clear benefit of taking risk preferences into account. While several psychiatric disorders are characterized by exhibiting extreme attitudes toward taking risks, most clinical studies that have examined impulsivity in these populations have not simultaneously assessed risk preference and have not done so in an incentive-compatible manner. For example, an implication of doing so could be that differences in discounting could be underestimated if in fact there are larger differences in risk preferences, e.g. if the control sample is significantly more risk averse than the clinical sample. Similarly, differences in risk preferences could be interpreted as differences in discounting. A few psychological studies in problem gamblers and alcoholics have performed both measures of temporal and probability discounting [79, 80]. Although these studies employ tasks very similar to our RA task for the estimation of probability discounting rates, they have not explicitly resolved the curvature of the utility function and therefore have not corrected for it in their estimation of discount rates. This would prove fairly easy to do using the methodology we propose here. The impact of ignoring risk preferences may reach beyond studies of clinical populations. In the social decision-making field for example, studies have suggested that discounting may be related to lack of cooperation [81] and to the willingness to punish other free-riders and non-cooperators [82, 83]. However, few of these studies have controlled for risk preferences, which may be important given that these seem to also correlate with individuals’ willingness to cooperate [84].

Temporal discounting is considered to be one of the many dimensions of the impulsivity personality construct. In the psychology literature, risk-seeking aspects of personality are often included in descriptions of impulsivity but there have been many attempts to separate these dimensions. Our approach results in the decorrelation of risk preference and time preference parameters (Fig 3) and allows for these parameters to reflect different aspects of decision-making and personality. We believe this separation could result in discount parameter estimates that may be more meaningful than those obtained using traditional methods. We propose a methodology that better resolves individual estimates of impulsivity and that could be easily incorporated in the growing literature on impulsivity by researchers interested in individual differences and behavioral phenotyping in clinical samples.

## Supporting information

S1 Fig Choice set space and example subject’s choices. A) Visualization of the ITC task trial space. Each dot is a trial composed of an offered immediate (no delay) monetary amount, a larger monetary amount to be delivered with a delay, and the delay to its delivery (in days). Shaded plains correspond to trials where the immediate monetary amount is the same, B) The same trial space displaying the choices made by an example subject. Blue dots correspond to the trials where the subject chose the immediate payment option and pink dots correspond to the trials where the subject chose the delayed payment. The boundary between pink and blue dots reflects the location of the indifference points in the space. Shaded plains correspond to trials where the immediate monetary amount is the same.
(TIF)

S2 Fig Model recovery analysis. Synthetic datasets were generated from each of the four models, LH, NLH, LE and NLE and then fitted with each model. Each cell in this matrix represents: AIC Fitted model—AIC True model. This difference equals 0 when the data generated from a model is fit with the same generative model. The larger the difference the worse the fitted model’s performance with respect to the generative model. Model LH is not a very discriminative model, models LE and NLE are especially bad a discriminating between each other. Out of the four models, model NLH is the most discriminative and the only that is significantly superior in goodness-of-fit to all other ones.
(TIF)

S3 Fig Cross comparison of all models. Cells indicate medians and 95% CI of bootstrapped log likelihood (LL) score differences. A positive median (in red) indicates that the model in the corresponding row had a higher score (better fit) than the model in the corresponding column.
(TIF)
