# Supplementary figures and images for: Risk preferences impose a hidden distortion on measures of choice impulsivity
Source: PLoS One. 2018 Jan 26;13(1):e0191357. doi: 10.1371/journal.pone.0191357 (PMC5786295; doi:10.1371/journal.pone.0191357)

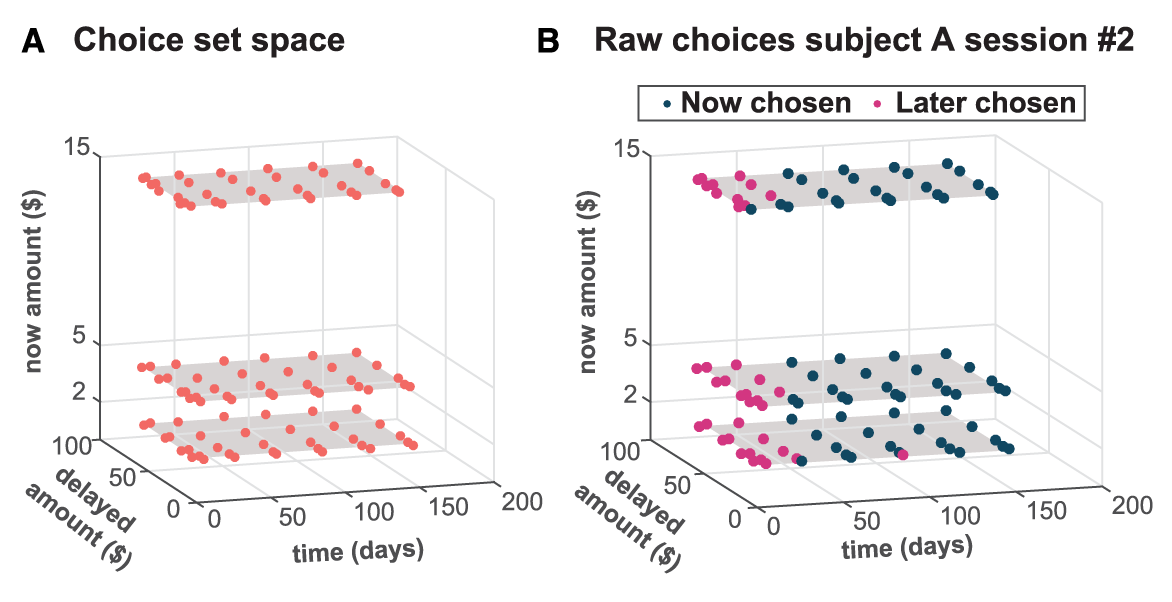

Supplement: S1 Fig — A) Visualization of the ITC task trial space. Each dot is a trial composed of an offered immediate (no delay) monetary amount, a larger monetary amount to be delivered with a delay, and the delay to its delivery (in days). Shaded plains correspond to trials where the immediate monetary amount is the same, B) The same trial space displaying the choices made by an example subject. Blue dots correspond to the trials where the subject chose the immediate payment option and pink dots correspond to the trials where the subject chose the delayed payment. The boundary between pink and blue dots reflects the location of the indifference points in the space. Shaded plains correspond to trials where the immediate monetary amount is the same. (TIF) [file pone.0191357.s001.tif]

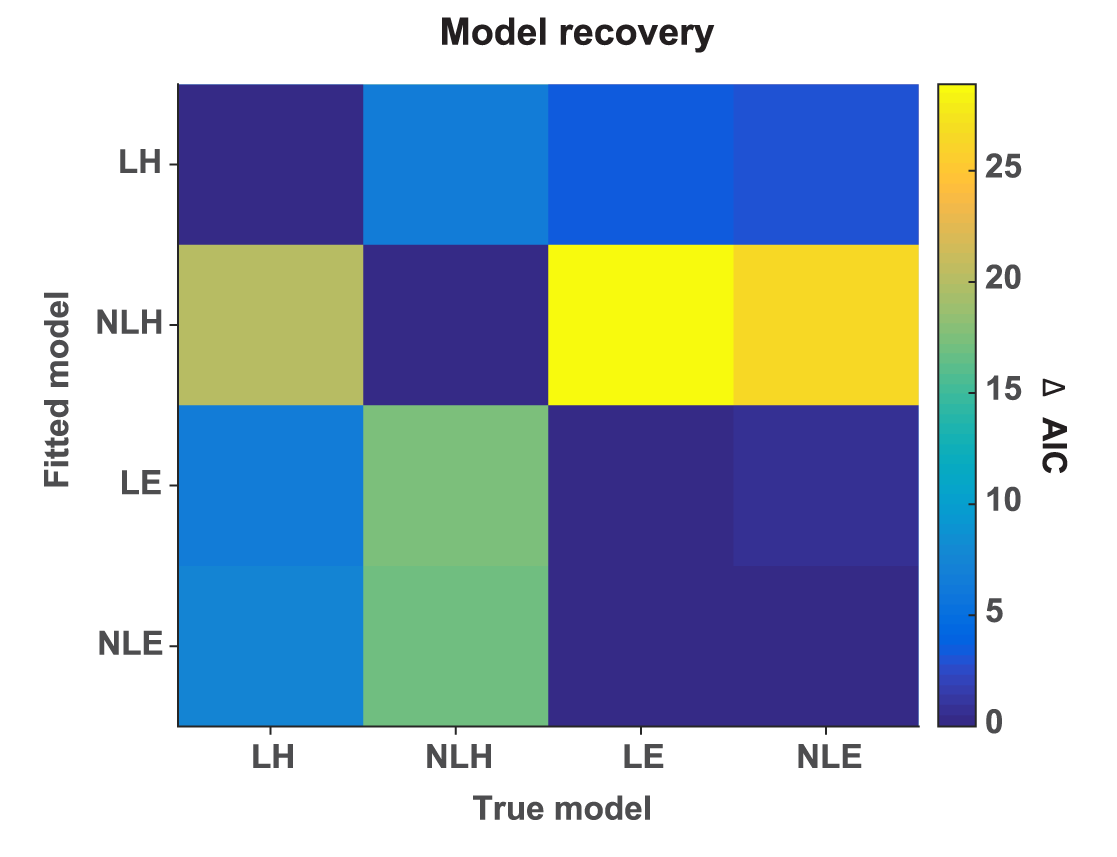

Supplement: S2 Fig — Synthetic datasets were generated from each of the four models, LH, NLH, LE and NLE and then fitted with each model. Each cell in this matrix represents: AIC Fitted model—AIC True model. This difference equals 0 when the data generated from a model is fit with the same generative model. The larger the difference the worse the fitted model’s performance with respect to the generative model. Model LH is not a very discriminative model, models LE and NLE are especially bad a discriminating between each other. Out of the four models, model NLH is the most discriminative and the only that is significantly superior in goodness-of-fit to all other ones. (TIF) [file pone.0191357.s002.tif]

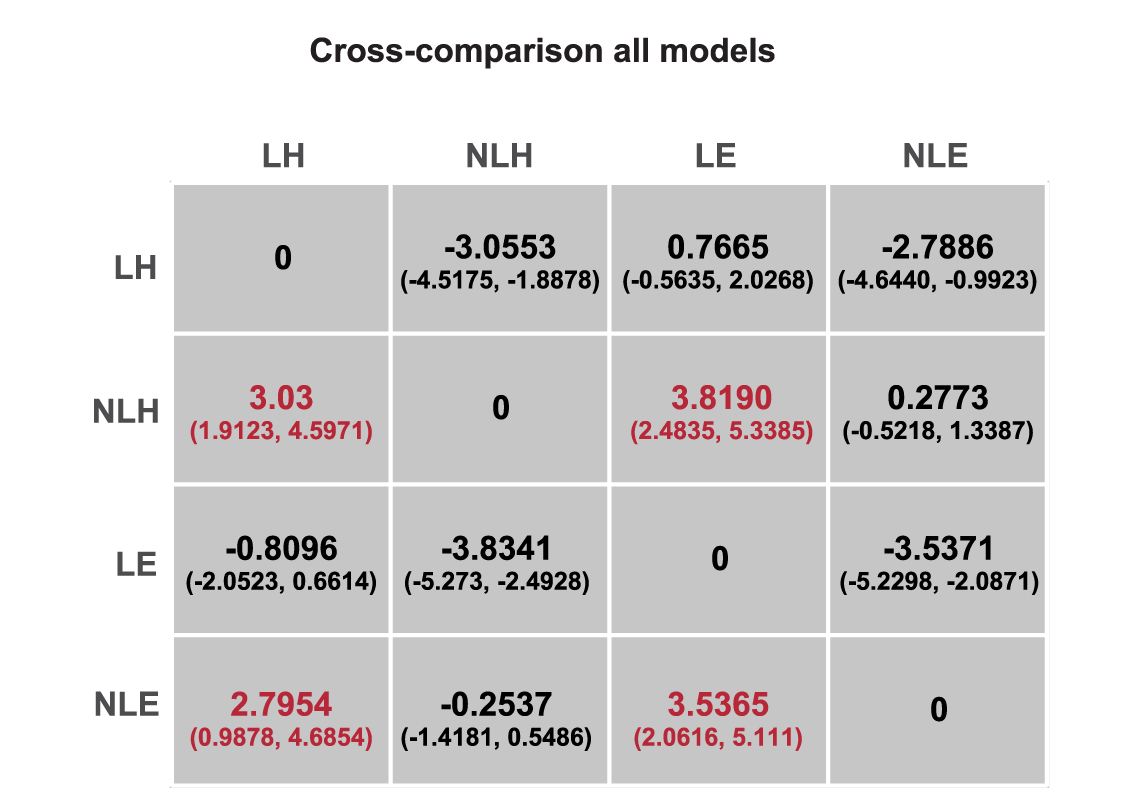

Supplement: S3 Fig — Cells indicate medians and 95% CI of bootstrapped log likelihood (LL) score differences. A positive median (in red) indicates that the model in the corresponding row had a higher score (better fit) than the model in the corresponding column. (TIF) [file pone.0191357.s003.tif]
